# Short screening tools for risky drinking in Aboriginal and Torres Strait Islander Australians: modified AUDIT-C and a new approach

**DOI:** 10.1186/s13722-019-0152-6

**Published:** 2019-07-01

**Authors:** K. S. Kylie Lee, James H. Conigrave, Scott Wilson, Jimmy Perry, Sarah Callinan, Robin Room, Tanya N. Chikritzhs, Tim Slade, Noel Hayman, Geoffrey Leggat, Katherine M. Conigrave

**Affiliations:** 1 NHMRC Centre of Research Excellence in Indigenous Health and Alcohol, Discipline of Addiction Medicine, Indigenous Health and Substance Use, Faculty of Medicine and Health, The University of Sydney, King George V Building, Level 6, 83-117 Missenden Road, Camperdown, NSW 2050 Australia; 2 Centre for Alcohol Policy Research, La Trobe University, Bundoora, VIC Australia; 3 Aboriginal Drug and Alcohol Council (ADAC), Underdale, SA Australia; 4 Health Sciences, National Drug Research Institute, Curtin University, Perth, WA Australia; 5 National Drug and Alcohol Research Centre, University of New South Wales, Sydney, NSW Australia; 6 Southern Queensland Centre of Excellence in Aboriginal and Torres Strait Islander Primary Health Care (Inala Indigenous Health Service), Inala, QLD Australia; 7 School of Medicine, Griffith University, Brisbane, QLD Australia; 8 School of Medicine, University of Queensland, Saint Lucia, QLD Australia; 9 Sydney Local Health District, Royal Prince Alfred Hospital, Drug Health Services, Camperdown, NSW Australia

**Keywords:** Aboriginal, Torres Strait Islander, Alcohol, Screening, Alcohol use disorder, AUDIT-C, Finnish method, Consumption

## Abstract

**Background:**

Alcohol consumption among Indigenous Australians can involve a stop-start pattern of drinking, with consumption well above recommended guidelines on each occasion. Such intermittent drinking patterns can make screening for risky drinking difficult. This study evaluates the ability of several short alcohol screening tools, contained in the Grog Survey Application, to detect short- or long-term risky drinking as defined by Australian guidelines. Tested tools include a modification of Alcohol Use Disorders Identification Test-Consumption (AUDIT-Cm).

**Methods:**

Alcohol consumption was assessed in current drinkers in the past year (n = 184) using AUDIT-Cm and using the last four drinking occasions (Finnish method). Sensitivity and specificity were assessed relative to the Finnish method, for how AUDIT-Cm score (3 + for women, 4 + for men), and how subsets of AUDIT-Cm questions (AUDIT-1m and AUDIT-2m; and AUDIT-3mV alone) were able to determine short- or long-term risk from drinking. Responses to AUDIT-Cm were used to calculate the average standard drinks consumed per day, and the frequency at which more than four standard drinks were consumed on single occasions. Finally, shorter versions of the Finnish method (1, 2, or 3 occasions of drinking) were compared to the full Finnish method, by examining the percentage of variance retained by shorter versions.

**Results:**

AUDIT-Cm has a high sensitivity in detecting at-risk drinking compared with the Finnish method (sensitivity = 99%, specificity = 67%). The combination of AUDIT-1m and AUDIT-2m was able to classify the drinking risk status for all but four individuals in the same way as the Finnish method did. For the Finnish method, two drinking sessions to calculate drinks per drinking occasion, and four to calculate frequency resulted in nearly identical estimates to data on all four of the most recent drinking occasions (r^2^ = 0.997).

**Conclusions:**

The combination of AUDIT-1m and AUDIT-2m may offer advantages as a short screening tool, over AUDIT-3mV, in groups where intermittent and high per occasion drinking is common. As an alternative to the full Finnish method, the quantity consumed on the last two occasions and timing of the last four occasions may provide a practical short screening tool.

**Electronic supplementary material:**

The online version of this article (10.1186/s13722-019-0152-6) contains supplementary material, which is available to authorized users.

## Background

There are similarities in patterns of alcohol consumption in Indigenous peoples in Australia and other colonised countries (e.g. New Zealand and Canada) [1–3]. Alcohol use by some individuals can be characterised as intermittent drinking that is well above national recommended guidelines for single occasions [1–3]. This raises difficulties when screening for risky drinking in these countries. For example, in Australia, there are concerns that current approaches used to screen or assess alcohol consumption are not well-suited for Australia’s First Peoples [4]. Similar concerns have been expressed in relation to assessment of drinking among Inuit, First Nation or Métis Canadians [5].

In survey settings, standard approaches, such as quantity-frequency or graduated quantity-frequency can be difficult to apply in populations with stop-start drinking patterns [5, 6]. In clinical settings, alcohol screening can be challenging due to potential sensitivity of the topic [7]. Also, alcohol may be one of a range of competing priorities for clinician and client, in a population with often numerous physical, mental and/or social presenting issues [8]. In Australia, less than 50% of individuals with unhealthy drinking are detected even in general practices serving the general population [9]. The percentage is similar in Aboriginal and Torres Strait Islander Community Controlled Health Services (ACCHSs) [10].

The Finnish method for assessing consumption [11] asks about the timing and quantity consumed in the last four drinking occasions [11]. It can be delivered in a conversational approach that may be suited to the storytelling traditions of Indigenous peoples [12].

The first three consumption items of the Alcohol Use Disorders Identification Test (AUDIT-C) form one of the most commonly used screening tools globally [13]. AUDIT-C is comprised of three items: AUDIT question 1 (AUDIT-1): “How often do you have a drink containing alcohol?”; AUDIT question 2 (AUDIT-2): “How many standard drinks do you have on a typical day when you are drinking?; and AUDIT question 3 (AUDIT-3): “How often do you have six or more standard drinks on one occasion?”.

Although AUDIT-C has been studied in Indigenous Australians more than other available screening tools [9], no validation studies have been conducted of its suitability with Australia’s First Peoples, Māori New Zealanders or Canadian Inuit, First Nation or Métis. In an effort to standardise and improve alcohol screening in Australia, AUDIT-C was made a national key performance indicator in 2017 by the Australian government for all ACCHSs [14]. But there may be difficulties with using AUDIT-C with Australia’s First Peoples. In particular, AUDIT-C assumes a usual drinking pattern and requires conversion of consumption to Australian ‘standard’ drinks [4]. This can be complex in any population, but more so when drinks are poured into non-standard containers for sharing (e.g. into empty soft drink bottles) [7]. Another short alcohol screening tool that has been used in Indigenous Australian settings is a modified AUDIT-3 (to assess how often an equivalent of 60 + grams of ethanol is consumed) [4]. However, data on the validity and accuracy of this tool is limited [4]. More also needs to be done to make screening easier for survey participants, clinic patients and clinicians.

To help Indigenous Australians describe their drinking, we developed and evaluated a tablet computer-based application to assess and record alcohol consumption [5]. The Grog Survey Application (App) asks about an individual’s last four drinking occasions (an adaptation of the ‘Finnish method’) [11] with elements of Timeline Followback [15]. The App uses data from the last four occasions to estimate how much people drink (average consumption, and grams per drinking day) and frequency of drinking [12]. This approach is likely to capture irregular or intermittent patterns of drinking better than tools which assume a regular frequency of drinking [12]. The App also provides a visual and interactive way for individuals to indicate what they drink—selecting from photographs of alcohol products, container types; and indicating fullness of containers and sharing of alcohol [16]. The App includes adapted versions of AUDIT-1 and AUDIT-3 (AUDIT-1m; AUDIT-3m visual [AUDIT-3mV]). An equivalent to AUDIT-2 (usual amount of drinking per occasion; AUDIT-2m) is calculated from the quantity of consumption recorded on the App by taking an average of the consumption on each of the last four drinking occasions.

Using a clinical interview performed by an Aboriginal health worker as a reference standard, the Grog Survey App performed well in measuring risky drinking (defined using Australian National Health and Medical Research Council [NHMRC] guidelines; Sensitivity 93%, Specificity 70% for short-term risk; Sensitivity 71%, Specificity 69% for long-term risk; when examining the same time frame) [17]. The App’s estimate of alcohol consumption also demonstrated good test–retest reliability (r = .81 within one week) [12].

While the Grog Survey App provides an acceptable [16] and valid [12] measure of drinking among Indigenous Australians, it took on average 10 min to administer. In both clinical settings and surveys there is a need for short, simple, validated screens for unhealthy alcohol use. Most ACCHSs around Australia now have AUDIT-C on their clinical practice software, and total score is calculated to indicate risk. However, it is necessary to look at subsets of the AUDIT-C items to understand whether that risk is comprised of short-term risk (e.g. injuries) or long-term risks (e.g. hypertension, cancers) or both.

Using data from the larger study on the development and evaluation of the Grog Survey App, we now evaluate the ability of short alcohol screening tools, contained within the App survey, to detect short-term or long-term risky drinking as defined by Australian NHMRC guidelines [17]. The analysis presented in the current paper examines the accuracy of AUDIT-C modified on the survey App, and subsets of AUDIT-C (modified versions of AUDIT-1 plus AUDIT-2; and AUDIT-3). The reference standard was the risk status as determined by the Finnish method collected via the survey App. Finally, we consider the effectiveness of asking shorter versions of the last four occasions (Finnish) method as a potential shortened screen in comparison to the full method.

## Methods

Study methods were designed by investigators in consultation with the Aboriginal Drug and Alcohol Council of South Australia; the Aboriginal Drug and Alcohol Network, representing Aboriginal alcohol and other drug workers in New South Wales (NSW); and the Aboriginal Health Council of South Australia (AHCSA), the peak body for ACCHSs in South Australia. Ethical approval was obtained from ACHSA and from Metro South Health Human Research Ethics Committee in Queensland.

### Recruitment

Stratified convenience sampling was used—we aimed to recruit: 20 non-drinkers, 40 non-dependent drinkers and 40 dependent drinkers in each of two state. Aboriginal field research assistants set up participants on iPads to complete the survey App. Research assistants recruited based on observation/anecdotal knowledge of the drinking category that potential participants would be in. Then at the end of each day all iPads were synchronised to the University of Sydney encrypted server and the App dashboard provided an update of the number of people in each category, to inform the next days’ recruitment efforts. Participants were classified as non-drinkers by the survey App if they reported not consuming alcohol in the preceding 12 months. To classify dependent and non-dependent drinkers, participants were asked on the survey App to rate the frequency of three alcohol dependence criteria (ICD-11) [7, 18]. Participants were classified as non-dependent if they rated these experiences as never occurring. Conversely, for the purpose of sampling, any participant who rated that these dependence symptoms occurred at any frequency in the past 12 months, were considered dependent.

Most of the analyses involved in validating [12] and shortening the scale used in the pilot study require little statistical power. For instance, for the reliability analysis, in order to have sufficient power (80%) to identify a correlation of 0.4 where *r* = 0.8, and ɑ = .05 a sample size of 46 is required (calculated using the ‘pwr’ package in R). Greater sizes were sought to try to allow analysis of differences between urban and remote sites, and because of anticipated challenges in ensuring complete data collection. In urban Queensland (QLD), recruitment was based in an Indigenous primary health care service and surrounding community (August–November 2016). In South Australia (SA), recruitment centred on a regional ACCHS and a remote Aboriginal community controlled drug and alcohol day centre (a non-residential drop-in service; two periods in August–September 2016 and April–May 2017). Individuals were eligible for inclusion if they self-identified as being Aboriginal or Torres Strait Islander and were 16 years or older. Exclusion criteria included obvious intoxication. Non-drinkers (defined as individuals who have not consumed alcohol in the last 12 months) were excluded from analyses in the current paper.

### Data collection and instruments

#### Grog Survey App

All survey items were delivered using the ‘Grog Survey App’. Broadly, the App presents questions on demographics, alcohol consumption (10 items), alcohol dependence (3 items based on ICD-11 [19]), harms to self or others, treatment access and participants’ feedback on using the App. An initial consultation workshop, combined with an iterative consultation process was used to develop the App. Weekly or more frequent advice was sought over a 12-month period from Indigenous and non-Indigenous clinicians and other health professionals, researchers and Indigenous community members nominated by their local community controlled health service [7].

**Fig. 1:**
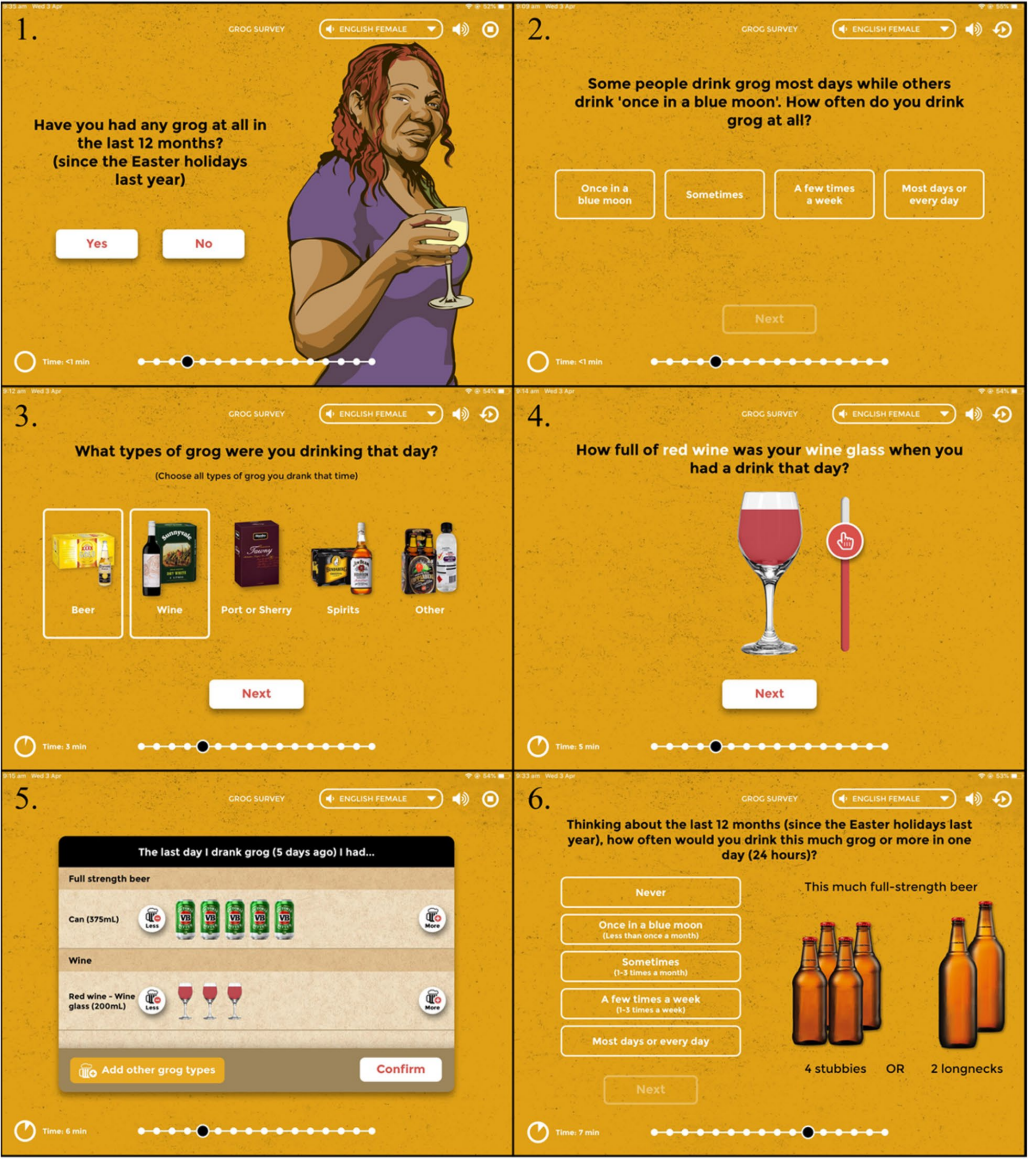
Items from the Grog Survey App used to ask the first three questions of the Alcohol Use Disorders Identification Test (AUDIT-C). 1–2 Screens used to ask AUDIT-1m (based on AUDIT question 1). 3–5 Selection of the screens used to ask about each of the last four drinking occasions (Finnish method). AUDIT-2m (quantity of drinking) was derived from the data on the last four drinking occasions collected on the App. 6. Screen used to ask AUDIT-3mV (a modification of AUDIT question 3). This uses the threshold of approximately 50 g of pure alcohol in line with definitions of short-term risk of drinking based on Australian National Health and Medical Research Council drinking guidelines. An image dynamically appears that is approximately equivalent to five Australian standard drinks (50 g) of the alcohol type most often consumed by each participant based on their last four drinking occasions

The App includes: culturally appropriate questioning style and gender-specific voice and images; ‘translation’ to colloquial English and (for audio) to the local Indigenous language (Pitjantjatjara); interactive visual approaches to estimate quantity of drinking; images of specific brands of alcohol, rather than abstract description of alcohol type (e.g. ‘spirits’); images of commercial and make-shift drinking containers (e.g. empty soft drink bottles); option to estimate consumption based on the individual’s share of what the group drank; key time points to explain time references for communities that do not routinely use calendars (e.g. ‘since last New Year’ to help explain ‘in the past 12 months’; see Fig. 1).

The App is able to detect 93% of those who were found to be at short-term risk of harms in a clinical interview conducted by an Aboriginal health professional (specificity: 70%) [12]. In this testing in regional/remote South Australia and urban Queensland, the App was also found to be highly acceptable [16]. Participation in the survey and its built in tailored brief intervention on drinking was found to prompt reflection on drinking in up to half the participants, based on spontaneous comments to the research assistants [16].

#### Alcohol consumption

After asking whether individuals consumed any alcohol at all in the past 12 months, the Finnish method and AUDIT-1m were each asked independently to estimate frequency of drinking. Australian standard drinks (10 g ethanol) consumed on each of the last four drinking occasions were estimated using a visual and interactive approach on the App, and responses to AUDIT-2m were derived from this. The frequency at which participants consumed at levels associated with risk of short-term harms (more than four standard drinks per occasion; 17) was asked using a modified, visual form of AUDIT-3 (AUDIT-3mV; Table 1).

**Table 1:** Variants of the Alcohol Use Disorders Identification Test-Consumption [AUDIT-C] items that were used on the Grog^a^ Survey App

Name of survey item	Original item wording	Our wording	Original response categories	Our response categories	Comments
‘Preparatory’ question asked before AUDIT-1m	–	Have you had any grog at all in the last 12 months (since Easter^b^ last year)?	–	Yes No	Used as a gentle introduction to the alcohol questions
‘Check’ question asked if response to ‘preparatory’ question is ‘no’	–	Can I check, in the last 12-months (since Easter^b^ last year), at special events like sporting carnivals, weddings or funerals, have you had any grog at all?	–	Yes, I’ve had some grog in the last 12 months No, I had no grog at all in the last 12 months	This was asked because of a clinical observation that respondents sometimes assume the questionnaire is only asking about ‘heavy’ or regular drinking
AUDIT-1m Question 1 modified	How often do you have a drink containing alcohol?	Some people drink grog most days while others drink ‘once in a blue moon’^c^. How often do you drink any grog at all?	Never Monthly or less 2–4 times a month 2–3 times week 4 or more times a week	Once in a blue moon (less than a month) Sometimes (1–3 times a month) A few times a week (2–3 times a week) Most days or every day	Only respondents who said they have consumed alcohol in the last 12 months (in the preparatory and check question above) were asked AUDIT-1m. Hence, a ‘never’ option was not provided
AUDIT-2m Question 2 modified	How many drinks containing alcohol do you have on a typical day when you are drinking?	[The usual amount of drinking alcohol per occasion was derived from each detailed response provided on what individuals consumed on their last four drinking occasions (“Finnish” method)]	1 or 2 3 or 4 5 or 6 7, 8, or 9 10 or more	[Original response categories were applied to the continuous data on drinking]	All respondents who were asked AUDIT-1m were then asked AUDIT-2m Derived from last four occasions data that was collected on the App
AUDIT-3mV Question 3 modified and presented visually	How often do you have six or more standard drinks on one occasion?	Thinking of the last 12-months [Since Easter last year]^a^, how often Would you drink [this much grog or more] in 1 day (24 h)?	Never Less than monthly Monthly Weekly Daily or almost daily	Never ‘Once in a blue moon’ (less than once a month) Sometimes (1–3 times a month) A few times a week (1–3 times a week) Most days or every day	All respondents who were asked AUDIT-1m and AUDIT-2m were then asked AUDIT-3mV Dynamically shows an image equivalent of 5 standard drinks (50 g ethanol) of the alcohol type most often consumed by each participant (based on the last four drinking occasions)

^a^Australian colloquial word for ‘alcohol’

^b^Example of reference points used to anchor answers reflecting on ‘in the last 12-months’ time period. This dynamically changes according to the time of year that the survey is completed. For example, other reference points include New Year and commonly known football grand finals

^c^Australian colloquial word for ‘rarely’

- Finnish method

Participants reported on their four most recent drinking occasions in the last 12 months. Participants reported when these occasions occurred, and how much alcohol they consumed on each. Rather than report on standard drinks, participants selected pictures of the alcohol product consumed, the container they drank from, and how full the container was with alcohol [7]. The App then converted these responses to Australian standard drinks.

- Modifications of AUDIT-C

As described elsewhere [7] the AUDIT-C questions and response options were converted to colloquial English to suit the Indigenous Australian context. For example, AUDIT-1 was reworded to: “Some people drink grog most days while others drink ‘once in a blue moon’. How often do you drink any grog at all?” (AUDIT-1m). As described above and below, AUDIT-2 was derived from the responses to the Finnish method (AUDIT-2m). AUDIT-3mV used a visual image of commonly used containers equivalent to five standard drinks (e.g. four cans of full strength beer). The image displayed used the type and brand of alcohol that the respondent reported consuming.

Responses to the AUDIT-1m and AUDIT-3mV are given on a five-point ordinal frequency scale ranging from “Never” to “Most days or every day”. As there was not a broad understanding of what a standard drink is in the target communities, and because in many remote communities the concept of ‘usual’ consumption may not exist [7], AUDIT-2m responses were derived from usual quantity consumed as estimated by the Finnish method. The continuous data on consumption were re-coded into the five AUDIT-2 response categories (a score of 0 indicates 1–2 drinks; 1, 3–4; 2, 5–6; 3, 7–9; 4,10 or more drinks, respectively).

### Analysis

All analyses were performed in R [20]. Sensitivity and specificity analyses were calculated with the ‘epiR’ package [21] (version 0.9-99). Correlations were calculated with the ‘stats’ package (version 3.5.3), included with base R. Only individuals who had consumed any alcohol in the past year (n = 184) were included in analyses. Using alcohol consumption data collected by the Finnish method on the App, a person was considered at risk from their drinking if their consumption exceeded the NHMRC guidelines [17]—that is, if they consumed an average of more than two standard drinks per day (long-term risk; guideline 1); or more than 4 standard drinks on any single drinking day (short-term risk; guideline 2).

As previously described [12], with the Finnish method, the timing of the last four drinking occasions in the past year was used to calculate drinking frequency (occasions per month). Data on quantity per occasion was used to calculate standard drinks per drinking occasion, and amount and timing of drinking occasions was used to estimate average drinks per day.

We then established how the short screening tools performed in comparison with risk status as determined using the Finnish method. Participants were classified at risk by their AUDIT-Cm score if their total score was equal to three or more for women, and four or more for men [22]. These are the thresholds recommended by the Australian government for use within ACCHSs.

We then assessed the accuracy of shorter versions of both these measures to determine either short-term or long-term risk from drinking in comparison with the full Finnish method. We examined the combination of AUDIT-1m and AUDIT2 m; and AUDIT-3mV alone. The midpoint of the AUDIT-1m and AUDIT-3mV response category selected was used to give a frequency (per day; so that weekly drinking is 0.14 times per day). Similarly, for AUDIT-2m, in order to mimic the performance of this test when delivered using response categories, the mid-point of the first four response categories was used to calculate the average drinks consumed per drinking occasion for those responses. As there is no upper bound for the top category (10 + drinks), 10 standard drinks was used.

Short-term risk was estimated by the combination of AUDIT-1m and AUDIT-2m if participants consumed more than four standard drinks per occasion and had consumed alcohol at least once in the last 12 months. Similarly, participants were classed as being at short-term risk by AUDIT-3mV if they indicated drinking more than four drinks on an occasion at least once in the past year.

Long-term risk was estimated with the AUDIT-1m and AUDIT-2m by multiplying the frequency of drinking (mid-point of response category of AUDIT-1m) by the average standard drinks per day (mid-point of response category for AUDIT-2m). Long-term risk was estimated with the AUDIT-3mV by multiplying five standard drinks (as this question asked about drinking 5 + drinks) by the mid-point of the frequency response selected for that level of drinking. We then compared the risk classifications of these two short forms to classifications made by the Finnish method in a sensitivity and specificity analysis (with the Finnish method as a reference standard). In order to calculate the sensitivity and specificity of the AUDIT-Cm, the table function, from base R, and the ‘epi.tests’ function, from the epiR package, were used together. A cross-tabulation of risk status was produced which compared risk status derived from responses to the AUDIT-Cm with responses derived from the Finnish method. This cross-tabulation was then supplied to the ‘epi.tests’ function in order to obtain sensitivity, specificity and their respective confidence intervals.

Finally, we used Pearson correlations (*r*) to demonstrate accuracy of shorter versions of the Finnish method in comparison to the full Finnish method, which uses the last four drinking occasions. We examined use of smaller numbers of drinking occasions (the last 1, 2 or 3) to estimate the average number of standard drinks per occasion, and the frequency of occasions. Correlations were squared in order to calculate r^2^ (the percentage of variance retained) by the shorter versions.

## Results

### Participants

**Fig. 2:**
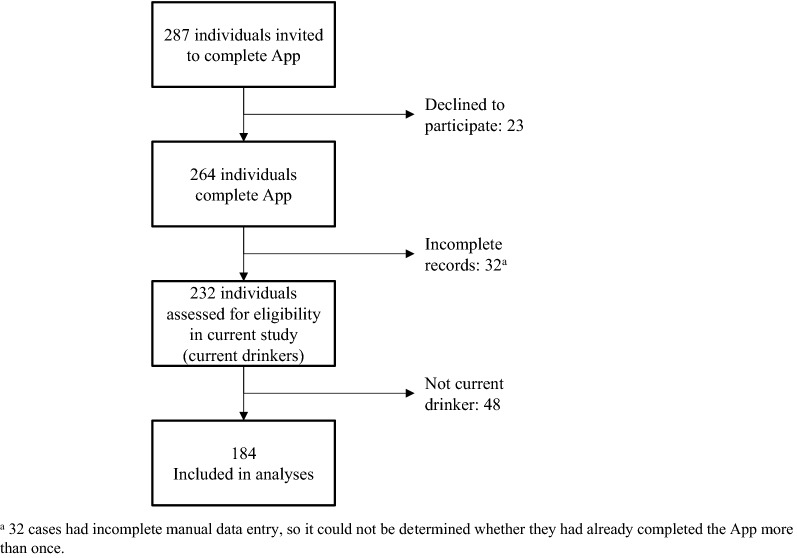
Sampling flow of participants recruited to this study

Participants were 184 Aboriginal and Torres Strait Islander peoples who had consumed alcohol in the past 12 months (Fig. 2). They had a mean age of 37.5 years (*SD* = 13.4). Of these, 28 (15.2%) were from regional areas, 66 from remote (35.9%) and 90 from urban centres (48.9%). Most (n = 137; 74.5%) had completed high school up to at least Year 10.

### Description of drinking risk in the sample, using different measures

The Finnish method (the reference standard) found a high number (n = 175; 95.1%) of drinkers were at risk from their drinking in the past year (95.1% short-term risk; 44.0% long-term risk). A very similar number of drinkers (n = 177; 96.2%) were classified as at risk based on their total AUDIT-Cm scores (96.2% scoring ≥ 4 for a man, 96.2% ≥ 3 for a woman). In keeping with this, responses to AUDIT-2m show that most drinkers (77.7%) consumed 10 or more standard drinks per drinking occasion (Table 2).

**Table 2:** AUDIT-C item responses and scores for drinkers

Score	0	1	2	3	4
AUDIT-1m (frequency of drinking)	Never	Less than monthly	1–3 times per month	1–3 times a week	Most days
%	0	30.4	42.4	26.1	1.1
AUDIT-2m (usual number of drinks)	1–2	3–4	5–6	7–9	10+
%	2.7	4.3	3.8	11.4	77.7
AUDIT-3mV (Frequency of drinking 5 + drinks per occasion)	Never	Less than monthly	1–3 times per month	1–3 times a week	Most days
%	14.1	34.2	39.1	10.3	2.2

n = 184

### Accuracy of AUDIT-Cm and subsets to detect risky drinking

Using the Finnish method as a reference test, the sensitivity of the AUDIT-Cm was 99% [95% CI 96.9, 100.0] and specificity 67% [95% CI 29.9, 92.5] in detecting at-risk drinking (either short-term or long-term risk). AUDIT-Cm only disagreed with the Finnish method on the risk status of four participants.

Subsets of items from the AUDIT-Cm were used to classify participants into NHMRC short- and long-term risk categories. Together, AUDIT-1m and AUDIT-2m identified almost two-thirds of drinkers (n = 122, 66.3%) as being at short-term risk from drinking at least once a month and a quarter (n = 48, 26.1%) of drinkers as being at long-term risk from their drinking. AUDIT-3mV identified 95 (51.6%) participants as being at short-term risk from their drinking (5 + drinks per occasion) at least once per month and classified four participants at long-term risk (2.2%; Table 3).

**Table 3:** Percent classified at risk based on AUDIT-Cm, and subsets of AUDIT-Cm items

	Short-term risk	Long-term risk
AUDIT-1m and AUDIT-2m	66.3	26.1
AUDIT-3mV	51.6	2.2
AUDIT-Cm	70.7	26.6
Finnish method	95.1	44.0

n = 184. Short-term risk = consumption of more than four standard drinks on any occasion; long-term risk = consumption of an average of more than two drinks per day

Table 4 presents the sensitivity and specificity of AUDIT-Cm and subsets of its items to detect at-risk drinking in comparison to the Finnish method (the reference). AUDIT-Cm and its subsets performed better in detecting short-term risk than long-term. AUDIT 3 mV had a lower sensitivity in detecting short-term risk than did the combination of AUDIT-1m and AUDIT 2 m, despite similar specificity. AUDIT 3 mV had low sensitivity in detection of long-term risk from drinking (3.7% [95% CI 0.8, 10.4]), but had high specificity (99.0% [95% CI 94.7, 100.0]). Its sensitivity was significantly lower than that of AUDIT-Cm or the combination of AUDIT-1m and AUDIT-2m, despite similar specificity.

**Table 4:** Comparing AUDIT-C and its item subsets against the Finnish method

	Short-term risk		Long-term risk	
	Sensitivity	Specificity	Sensitivity	Specificity
AUDIT-1m and AUDIT-2m	69.7 (62.3, 76.4)	100.0 (66.4, 100.0)	46.9 (35.7, 58.3)	90.3 (82.9, 95.2)
AUDIT-3mV	53.7 (46.0, 61.3)	88.9 (51.8, 99.7)	3.7 (0.8, 10.4)	99.0 (94.7, 100.0)
AUDIT-Cm	73.7 (66.5, 80.1)	88.9 (51.8, 99.7)	48.1 (36.9, 59.5)	90.3 (82.9, 95.2)

n = 184. Short-term risk = consumption of more than four standard drinks on any occasion; long-term risk = consumption of an average of more than two drinks per day; 95% confidence intervals presented in brackets

### Potential shortening of the Finnish method

Finally, we explored if the Finnish method can be shortened to enable fewer than four drinking occasions to be asked about on the survey App without loss of accuracy. Table 5 presents the total variance (the squared correlation) between each of these shorter estimates of consumption, and the estimate derived from using all four sessions.

**Table 5:** The total variance explained by shorter versions of the Finnish method in comparison to the full Finnish method

		Number of occasions used to estimate frequency of drinking			
		1 (%)	2 (%)	3 (%)	4 (%)
Number of occasions used to estimate quantity per drinking occasion	1	95.9	95.9	96.1	96.6
	2	98.6	98.6	98.8	99.7
	3	98.6	98.6	98.8	99.6
	4	98.8	98.8	99.0	100.0

n = 184

Using two drinking sessions to calculate drinking intensity (drinks per drinking occasion) and four to calculate frequency resulted in estimates that were nearly identical to those obtained when collecting data on all four of the most recent drinking occasions (r^2^ = 0.997). In order to exclude the possibility that this was due to reporting where participants gave identical answers for each occasion, we excluded cases with zero variation in amounts consumed between occasions. Similar results were obtained (intensity calculated from two, and frequency derived from four sessions r^2^ = 0.994).

## Discussion

This study is one of only a small number [20, 21] to have tested the accuracy of short alcohol screening tools to detect risky drinking in Australia’s First Peoples, and to our knowledge in First Peoples worldwide. This study examined the extent to which subsets of AUDIT-Cm (AUDIT-1m plus AUDIT-2m; AUDIT-3mV), and shortened versions of the ‘last four occasions’ (Finnish) method contribute to assessment of drinking risk. In this sample, total AUDIT-Cm score and the Finnish method only disagreed on the risk categorisation of four participants. However our results raised questions about the suggested use of AUDIT-3 alone to assess drinking risk [23, 24]. In this sample, AUDIT-3mV performed less well than the combination of AUDIT-1m and AUDIT-2m, or the full AUDIT-Cm in detecting individuals as being at risk of short-term risk from their drinking (as classified by the Finnish method). AUDIT-3mV had a poor ability to detect long-term risk from drinking. Finally, we showed that in this sample of drinkers, asking about an individual’s last one or two drinking occasions could be an effective shortened screen for assessing drinking risk.

### Shorter versions of AUDIT-Cm

This stratified sample was comprised of individuals who drink intermittently (most commonly 1–3 times a month [42.4%]). But when they do drink they tend to consume large quantities. More than three-quarters (77.7%) of our subjects reported 10 or more Australian standard drinks on drinking occasions. While the current study over-sampled likely dependent drinkers, this pattern of infrequent heavy drinking occasions has been reported elsewhere for Indigenous Australians in several studies [2].

The combination of AUDIT-1m and AUDIT-2m detected a little over two-thirds of the drinkers who were identified as being at short-term risk from their drinking (using the Finnish Method as a reference standard) (sensitivity 69.7% [95% CI 62.3, 76.4], specificity 100% [95% CI 66.4, 100.0]). Adding AUDIT-3mV only slightly increased the sensitivity (sensitivity 73.7% [95% CI 66.5, 80.1], specificity 88.9% [95% CI 51.8, 99.7]). Accordingly, AUDIT-3mV may offer limited benefit in communities where there is typically a high number of drinks consumed per drinking occasion.

AUDIT-3 has been proposed as a single item screening tool for some populations [23]. However, in this sample, AUDIT-3mV alone detected just over half of those drinking at short-term risk (sensitivity 53.7% [95% CI 46.0, 61.3], specificity 88.9% [95% CI 51.8, 99.7]; using the Finnish method as reference test). It is not clear why this is the case, given the high number of drinks reported per drinking occasion. Consultation during the process of App development indicated that many Aboriginal people who drink in a stop-start manner may find it difficult to respond to a question on *usual* frequency of a drinking behaviour. It is less surprising that AUDIT-3mV performed poorly at detecting people who were at long term-risk from their drinking (sensitivity 3.7% [95% CI 0.8, 10.4], specificity 99% [95% CI 94.7, 100.0]), as the threshold of AUDIT-3mV (5 + drinks per occasion) is considerably higher than the Australian threshold for long-term risk (2 + drinks per day). Also, the relatively low frequency of drinking among this population results in a lower average consumption per day.

AUDIT-3mV was asked using a visual representation of the drinking threshold. In at least one other study [24] such a visual representation has been successfully employed to show this drinking threshold, using several common types of alcohol. However, as far as we are aware, this is the first time that a visual representation has been tailored to individual participants by a computer App so that it shows the threshold using the alcohol type most often consumed by that individual, (e.g. a beer drinker may see four cans of beer, a wine drinker would see four home-poured glasses of wine). This would be expected to have provided greater accuracy for AUDIT-3mV than its standard administration, where the client or the clinician needs to mentally convert that threshold to the type of preferred alcoholic beverage that a person drinks.

### Shortening the last four occasions (Finnish) method

We found that simply asking in detail about the quantity consumed on the last two occasions and about the timing of the last two drinking occasions was as good as asking about both quantity and timing for the last four drinking occasions (98.6% % of the variance was explained). Even the quantity from the last occasion and the timing of the last two occasions explained 95.9% of the variance explained by the full Finnish method. In keeping with this, Gregson and Stacey [25] found that the quantity consumed in the last drinking occasion, and the date of the last two drinking occasions was a useful estimate of drinking.

Any approach to estimating drinking can be difficult when drinking is stop-start (e.g. a person from dry community comes to town for 2 weeks and drinks daily while there), and the last two occasions approach has the potential to be inaccurate if the timing of those occasions is not typical of usual drinking frequency [11, 26]. In the current study, considering the timing of all four of the most recent drinking occasions reduces but does not eliminate this risk. The interactivity of a clinical interview allows these subtleties to be understood. In a tablet-computer platform, asking in detail about the quantity of the last two drinking occasions, and the timing of the last four (in the past year) would help to shorten the Grog Survey App while still retain accuracy (r^2^ = 0.997).

One important difference with our method of assessment that was not present in past studies [11, 25, 26] was the option for respondents to select the quantity consumed on their most recent occasion as the default quantity for prior occasions. Respondents could say that their quantity consumed was identical to the most recent occasion. Or they could use the drink types, containers and quantities from the most recent occasion as the starting point to describe their drinking on prior occasions, increasing or reducing quantities or changing drink types as needed to describe what had consumed. These options may have led to respondents providing estimates of drinking quantities in the last four occasions that were more similar to each other than in reality. However, these results (on the value of the last two occasions as predictors of drinking pattern over the last four occasions) were consistent after cases with zero variation between drinking occasions were excluded. This could reflect a somewhat stereotyped pattern of drinking, with the goal of intoxication, in our sample. Further study of drinking patterns in Australia’s First Peoples is needed.

### Study limitations

AUDIT-1m and AUDIT-3mV were asked as part of the larger questionnaire, and not in their standard order. We cannot assume that these questions will behave the same way if administered as standalone items. This requires further investigation.

The last four occasions (Finnish) method has been used as the reference standard because it was well correlated when compared with a clinical interview conducted by an Aboriginal health professional [12]. However, in the current analysis, data from the Finnish method is also used to determine the response categories of AUDIT2 m, hence creating some circularity. Despite this, given the typically high quantity consumed per drinking occasion in this sample (median of 16–18 standard drinks per drinking occasion in both the survey App and clinical interview), it would seem likely that most drinkers would still have been detected as risky if other methods had been used to record quantity.

Here AUDIT-2m (usual quantity of drinking) was derived from a visual approach, tailored to each individual to estimate consumption. This may have increased its accuracy over an approach where the client or interviewer is required to convert drinking to standard drinks. In a clinical or survey setting accuracy relies on the client and/or interviewer understanding standard drink sizes and being comfortable with arithmetic for conversion. Visual aids are likely to be needed to assist in this process. Furthermore, the App considered sharing of drinks, which is sometimes overlooked in a clinical setting [27, 28], resulting in accidental over-estimation of consumption.

It is not clear how generalisable these study findings would be to other indigenous populations beyond the study regions. The current sample over-sampled likely dependent drinkers. It also was conducted in relatively under-privileged regions (one regional and two remote), and respondents might be more prone to all-or-nothing drinking than a higher socio-economic status region. Further research is needed using a representative sample from two or more Indigenous Australian communities, to re-test these survey approaches and to describe the local prevalence of different drinking patterns (Additional file 1).

We are unable to provide the raw participant-level data used in the reported analyses. Data was collected from small Australian Aboriginal communities and we do not have ethical clearance to release these datasets. Complete code used for the analyses presented are available in Additional file 1.

## Conclusions

Screening for risky drinking is difficult to do in any population, but there can be added challenges in Indigenous, culturally diverse or disadvantaged populations. The combination of AUDIT-1m and AUDIT-2m may be a superior short screening tool, relative to AUDIT-3mV, in groups where intermittent and high per occasion drinking is common. Asking about quantity consumed on the last two occasions and the timing of the last four occasions appears less time consuming than the full Finnish method, but still retains the majority of its accuracy. In populations with high rates of short-term risk from drinking, a respectful discussion about drinking and education on how to reduce risk from drinking may be helpful for any individual who says that they drink.

## Additional file

**Additional file 1.** Complete code for the analyses presented in this study.

## Data Availability

Data for this project is stored at the University of Sydney based at Drug Health Service, KGV Building, Missenden Road, Camperdown New South Wales, 2050 Australia.
