# Supplementary material for: Short screening tools for risky drinking in Aboriginal and Torres Strait Islander Australians: modified AUDIT-C and a new approach
Source: Addict Sci Clin Pract. 2019 Jul 1;14:22. doi: 10.1186/s13722-019-0152-6 (PMC6600888; doi:10.1186/s13722-019-0152-6)
Supplement: Supplementary file 1 — Additional file 1. Complete code for the analyses presented in this study. [file 13722_2019_152_MOESM1_ESM.pdf]

Short screening tools for risky drinking in Aboriginal and Torres Strait Islander Australians: modified AUDIT-C and a new approach: results

Overview

This document presents the results used in the production of this paper. Please note the dataset is highly sensitive as it was collected from small Aboriginal communities. As such, due to ethical restrictions, it is withheld.

Set-up

libraries and sources

A note on packages

Please note that 'papertools' version 0.4.3.1 was used in formatting these results. This package was developed by one of the authors and is available open-source on github: https://github.com/JConigrave

To install and inspect the specific version used in this report, please run the following code:

```
devtools::install_github("JConigrave/papertools@0.4.3.1").
```

Load libraries and data

```
library(tidyverse)
```

```
## -- Attaching packages ----------------------------------- tidyverse 1.2.1 --
```

```
## v ggplot2 3.1.0      v purrr   0.3.2
## v tibble  2.1.1      v dplyr   0.8.0.1
## v tidyr   0.8.3      v stringr 1.4.0
## v readr   1.3.1      v forcats 0.4.0
```

```
## -- Conflicts -------------------------------------- tidyverse_conflicts() --
## x dplyr::filter() masks stats::filter()
## x dplyr::lag()    masks stats::lag()
```

```
levels = c("Never","Once in a blue moon (<1/month)","Sometimes (1-3 times/month)","A few times a week (
           "Most days or every day")

f = readRDS("grog_app_complete.RDS") %>%
  filter(drinker_a ==1) %>%
  mutate(dependence_boss_a = factor(dependence_boss_a, levels = levels),
         dependence_time_a = factor(dependence_time_a, levels = levels))
tr = f[f$sd_a == 0,] #separate cases where there was no variance in drinking.
```

function for median and IQRs

```
m_iqr = function(x,round =2){
  med = round(median(x , na.rm=T),round)
  iqr = round(IQR(x, na.rm=T), round)
  return(paste0(med," (",iqr,")"))
  }
```

function to obtain sessions for individals (FINNISH METHOD)

```
getSessions = function(i,
                       type = "a",
                       start = NULL,
                       finish = NULL,
                       drinking_only = F,
                       data = f) {
  if (type == "a") {
    #this chunk gets the sessions
    t = data.frame(matrix(ncol = 2, nrow = 4)) #create a data frame
    t[, 1] = as.numeric(data[i, paste("session_a", 1:4, sep = "")]) #sessions across the columsn
    t[, 2] = do.call("c", lapply(1:4, function(x)
      data[i, paste("sessiondate_a", 1:4, sep = "")][[x]])) #turn into vector
    names(t) = c("intake", "date")
    rownames(t) = paste("session", 1:4, sep = "")

    #this chunk adds in missing days
    y = na.omit(t) #grab the sessions
    if (nrow(y) > 0) {
      sessions = as.numeric(difftime(data$surveydate_a[i],
                                    min(y$date, na.rm = T), units = "days")) #store how many days data
      t = data.frame(matrix(ncol = 2, nrow = as.numeric(sessions))) #create an empty data frame
      names(t) = c("intake", "date") #name cols
      t$intake = 0 #set all days to 0 intake
      t$date = do.call("c", lapply(1:sessions, function(x)
        as.Date(min(
          y$date, na.rm = T
        )) - 1 + x)) #put in appropriate dates
      for (d in seq_along(y$date)) {
        #for every date in y, put the intake into the right day of t
        t[t$date == y$date[d], "intake"] = y$intake[d]
      }
    } else{
      sessions = 365 #if they didn't put any sessions in, that means they didn't drink for the last yea
      t = data.frame(matrix(ncol = 2, nrow = as.numeric(sessions))) #create an empty data frame
      names(t) = c("intake", "date") #name cols
      t$intake = 0 #set all days to 0 intake
      t$date = do.call("c", lapply(1:sessions, function(x)
        as.Date(data$surveydate_a[i]) - x)) #put in appropriate dates
    }
  }
  if (type == "i") {
    t = data.frame(matrix(ncol = 2, nrow = 27)) #create an empty dataframe
```

```
    names(t) = c("intake", "date") #with names intake and date
    idays = (paste("day", 1:27, sep = "")) #store a vector of the name variables
    rownames(t) = idays #put the days in the rownames
    t[, 1] = do.call("c", list(t(data[i, idays]))) # grab the day data and throw it in the intake colum
    t[, 2] = do.call("c", lapply(1:27, function(x)
      as.Date(data$surveydate_i[i]) - x)) # put the date in which they occured under date
    t = t[!is.na(t$intake), ] #I don't need those NA rows. get rid of them!
  }

  if (!is.null(start)) {
    t = t[which(t$date <= start & t$date >= finish), ]
  }

  if (drinking_only == T) {
    t = t[t$intake != 0, ]

  }
  return(t)
}
```

Classify risk function

```
classRisk = function(avg_drinks,max_drinks, cutpoints = c(4,2,10,5)){
  ##short term risk
  s.result = ifelse(max_drinks > cutpoints[1],"high","low")

  ##longterm risk
  l.result = ifelse(avg_drinks > cutpoints[2],"high","low")

  ##short term - severe
  ss.result = ifelse(max_drinks > cutpoints[3],"high","low")

  ##longterm - severe
  ls.result = ifelse(avg_drinks > cutpoints[4],"high","low")
  return(list(short = s.result,long= l.result,short_severe = ss.result,long_severe = ls.result))
}
```

Set up AUDIT variables

```
audit_levels = c(0,1,2,3,4)
#audit1...
f$audit_1= NA
f$audit_1[f$frequency_audit1_a == 0] = 0
f$audit_1[f$frequency_audit1_a == .5] = 1
f$audit_1[f$frequency_audit1_a == 2] = 2
f$audit_1[f$frequency_audit1_a == 8.69714285714286] = 3
f$audit_1[f$frequency_audit1_a == 23.9171428571429] = 4
f$audit_1 = factor(f$audit_1,audit_levels)
#audit2...
f$audit_2 = NA
```

```
f$audit_2[f$drinker_a == 0] = 0
f$audit_2[f$drinking_intensity_a <= 2] =0
f$audit_2[f$drinking_intensity_a > 2 & f$drinking_intensity_a <= 4 ] = 1
f$audit_2[f$drinking_intensity_a > 4 & f$drinking_intensity_a <= 6 ] = 2
f$audit_2[f$drinking_intensity_a > 6 & f$drinking_intensity_a <= 9 ] = 3
f$audit_2[f$drinking_intensity_a > 9] = 4
f$audit_2 = factor(f$audit_2,audit_levels)

#audit2_reverse
f$audit_2_reverse = NA
f$audit_2_reverse[as.character(f$audit_2) ==0] = 1.5
f$audit_2_reverse[as.character(f$audit_2) ==1] = 3.5
f$audit_2_reverse[as.character(f$audit_2) ==2] = 5.5
f$audit_2_reverse[as.character(f$audit_2) ==3] = 8
f$audit_2_reverse[as.character(f$audit_2) ==4] = 10

#audit3...
f$audit_3 = NA
f$audit_3[f$drinker_a == 0] = 0
f$audit_3[f$audit3_a == 0] = 0
f$audit_3[f$audit3_a == .5] = 1
f$audit_3[f$audit3_a == 2] = 2
f$audit_3[f$audit3_a == 8.69714285714286] = 3
f$audit_3[f$audit3_a == 23.9171428571429] = 4
f$audit_3 = factor(f$audit_3,audit_levels)


f$audit_c = lapply(f[,c("audit_1","audit_2","audit_3")], function(x){
  as.numeric(as.character(x))
}) %>%
  as.data.frame() %>%
  rowSums()
```

Clasify risk

```
f$audit_risk = ifelse(
  (f$gender_a == "male" & f$audit_c >= 4) |
  (f$gender_a == "female" & f$audit_c >= 3),"high","low"
) %>% factor(levels = c("high","low"))


f$finnish_risk = ifelse(f$short_a == "high" | f$long_a == "high","high","low")%>%
  factor(levels = c("high","low"))

list(table(f$audit_risk),
     table(f$finnish_risk))%>%
  do.call(rbind,.) %>%
  data.frame(row.names = c("audit_c","finnish")) %>%
  knitr::kable(booktabs = T, longtable = F, format = "latex")
```

	high	low
audit_c	177	7
finnish	175	9

Converting AUDIT-C responses to continuous variables.

```
##### ###COMPARING AUDIT 1-2 with 3
f$audit1_estimate = (f$frequency_audit1_a * f$audit_2_reverse) / 30.44
f$audit1_estimate[f$drinker_a == 0] = 0
f$audit3_estimate = (f$audit3_a*5)/30.44 #five is the standard drink amount exceeding guidelines

audit_mqr = tibble(audit_c = c("audit_1","audit_2","audit_3"))
audit_mqr$median_iqr = lapply(c("audit1_estimate","audit_2_reverse","audit3_estimate"),function(x){
  f[,x] %>%
    m_iqr}) %>%
  unlist()

audit_mqr %>%
  knitr::kable(booktabs = T, longtable = F, format = "latex")
```

audit_c	median_iqr
audit_1	0.66 (2.12)
audit_2	10 (0)
audit_3	0.33 (0.25)

Define risk categories for AUDIT-C items. Also create a function to get sens and spec quickly.

```
short1 = ifelse(f$frequency_audit1_a > 1 & f$audit_2_reverse > 4,"high","low")
long1 = ifelse(f$audit1_estimate > 2, "high","low")


short3 = ifelse(f$audit3_a > 1,"high","low")
long3 = ifelse(f$audit3_estimate > 2, "high","low")

short_auditc = ifelse(short3=="high"|short1 =="high","high","low")
long_auditc = ifelse(long1 == "high" | long3 == "high","high","low")
```

Sensitivity and specificity function

```
get_sens.spec = function(short,long){
  short = factor(short,levels = c("high","low"))
  ss.short = table(short, factor(f$short_a, levels = c("high","low"))) %>%
    epiR::epi.tests()
  ss.long = table(long, factor(f$long_a, levels = c("high","low"))) %>%
    epiR::epi.tests()

  get_se = function(obj){
```

```
    results = obj$elements
      sens = papertools::glue_bracket(results$se*100,
                                       results$se.low*100,
                                       results$se.up*100,
                                       round = 1)
  spec = papertools::glue_bracket(results$sp*100,
                                       results$sp.low*100,
                                       results$sp.up*100,
                                       round = 1)
  list(sens = sens, spec = spec)
  }

  short_sens = ss.short %>% get_se() %>% .$sens
  short_spec = ss.short %>% get_se() %>% .$spec

  long_sens = ss.long %>% get_se() %>% .$sens
  long_spec = ss.long %>% get_se() %>% .$spec



 out =cbind(short_sens,short_spec,long_sens,long_spec)
 colnames(out) = c("Sens","Spec","Sens","Spec")
 rownames(out) = NULL
 out
}
```

Results

Demographics features of the sample

```
demographic_table = list( # create a list with demographic results
  participants = nrow(f),
  `average age` = papertools::glue_bracket( # put SD in bracket
    mean(f$age_a, na.rm = T),
    sd(f$age_a, na.rm = T),
    brackets = c("(SD =  ", ")"),
    round = 2
  ),
  `regional n` = papertools::n_percent(f$remoteness, "Regional"),
  `remote n` = papertools::n_percent(f$remoteness, "Remote"),
  `urban n` = papertools::n_percent(f$remoteness, "Urban"),
  `yr_10 complete` = papertools::n_percent(f$school > 9,"TRUE")
) %>%
  as_tibble %>%
  gather %>%
  rename(variable = key)

demographic_table %>%
  knitr::kable(booktabs = T, longtable = F, format = "latex")
```

variable	value
participants	184
average age	37.46 (SD = 13.36)
regional n	28.00 (15.22%)
remote n	66.00 (35.87%)
urban n	90.00 (48.91%)
yr_10 complete	137.00 (74.46%)

Table 1. AUDIT-C item responses and scores for drinkers

```
table_1 = list(table(f$audit_1), table(f$audit_2), table(f$audit_3))%>%
  lapply(.,function(x) prop.table(x)*100)%>%
  do.call(rbind,.) %>%
  as.data.frame(row.names = c("audit_1","audit_2","audit_3"))%>%
  round(1)

table_1 %>%
 knitr::kable(booktabs = T, longtable = F, format = "latex")
```

	0	1	2	3	4
audit_1	0.0	30.4	42.4	26.1	1.1
audit_2	2.7	4.3	3.8	11.4	77.7
audit_3	14.1	34.2	39.1	10.3	2.2

Table 2. Percent classified at risk based on AUDIT-C, and subsets of AUDIT-C items

```
short1_table = table(short1) %>% prop.table
long1_table = table(long1) %>% prop.table
short3_table = table(short3) %>% prop.table
long3_table = table(long3) %>% prop.table
short_auditc_table = table(short_auditc) %>% prop.table
long_auditc_table = table(long_auditc) %>% prop.table

short_a_table = table(f$short_a) %>% prop.table
long_a_table = table(f$long_a) %>% prop.table

props = tibble(tool = c("AUDIT1&2", "AUDIT3", "AUDIT-C", "FINNISH"))
props$short = c(short1_table[["high"]],
                short3_table[["high"]],
                short_auditc_table[["high"]],
                short_a_table[["high"]])
props$long = c(long1_table[["high"]], long3_table[["high"]],
               long_auditc_table[["high"]], long_a_table[["high"]])

table2 = apply(props[, 2:3], 2, function(x)
  round(x * 100, 1)) %>% as.data.frame() %>%
  mutate(tool = c(c("AUDIT1&2", "AUDIT3", "AUDITC", "FINNISH"))) %>%
  dplyr::select(" " = tool, short, long)
```

```r
table2 %>%
  knitr::kable(booktabs = T,
               longtable = F,
               format = "latex")
```

	short	long
AUDIT1&2	66.3	26.1
AUDIT3	51.6	2.2
AUDITC	70.7	26.6
FINNISH	95.1	44.0

Risk by gender

We use the audit_C to classify risk, their are different cut-offs for males an d females.

females:

```r
list(prop.table(table(f$audit_risk[f$gender_a == "female"])),
     prop.table(table(f$finnish_risk[f$gender_a == "female"])))%>%
  do.call(rbind,.) %>%
  data.frame(row.names = c("audit_c","finnish")) %>%
  knitr::kable(booktabs = T, longtable = F, format = "latex")
```

	high	low
audit_c	0.9615385	0.0384615
finnish	0.9230769	0.0769231

males:

```r
list(prop.table(table(f$audit_risk[f$gender_a == "male"])),
     prop.table(table(f$finnish_risk[f$gender_a == "male"])))%>%
  do.call(rbind,.) %>%
  data.frame(row.names = c("audit_c","finnish")) %>%
  knitr::kable(booktabs = T, longtable = F, format = "latex")
```

	high	low
audit_c	0.9622642	0.0377358
finnish	0.9716981	0.0283019

Sensitivity and specificity of AUDIT vs Finnish Method risk (any risk)

```r
sens__spec_results = table(f$audit_risk,f$finnish_risk) %>%
  epiR::epi.tests() %>%
  .$elements

data.frame(Sensitivity = papertools::glue_bracket(sens__spec_results$se*100,
                                        sens__spec_results$se.low*100,
                                        sens__spec_results$se.up*100,
                                        round = 1),
       Specificity = papertools::glue_bracket(sens__spec_results$sp*100,
                                        sens__spec_results$sp.low*100,
                                        sens__spec_results$sp.up*100,
```

```
                                                     round = 1)) %>%
  knitr::kable(booktabs = T, longtable = F, format = "latex")
```

Sensitivity	Specificity
99.4 (96.9, 100.0)	66.7 (29.9, 92.5)

Table 3. Comparing AUDIT-C and its item subsets against the Finnish method

```
table_3 = list(
  get_sens.spec(short1,long1),
  get_sens.spec(short3,long3),
  get_sens.spec(short_auditc,long_auditc)) %>%
  do.call(rbind,.) %>%
  data.frame()
rownames(table_3) = c("AUDIT-1m and AUDIT-2m", "AUDIT-3mv", "AUDIT-c")
names(table_3) = c("short sensitivity","short specificity","long sensitivity","long specificity")

 table_3 %>%
  knitr::kable(booktabs = T, longtable = F, format = "latex")
```

	short sensitivity	short specificity	long sensitivity	long specificity
AUDIT-1m and AUDIT-2m	69.7 (62.3, 76.4)	100.0 (66.4, 100.0)	46.9 (35.7, 58.3)	90.3 (82.9, 95.2)
AUDIT-3mv	53.7 (46.0, 61.3)	88.9 (51.8, 99.7)	3.7 (0.8, 10.4)	99.0 (94.7, 100.0)
AUDIT-c	73.7 (66.5, 80.1)	88.9 (51.8, 99.7)	48.1 (36.9, 59.5)	90.3 (82.9, 95.2)

Function for shortening Finnish method

```
shorten = function(n_session, n_freq, data = f){
require(lubridate)
  max = c()
  intensity = c()
  average = c()
  frequency = c()
  short = c()
  long = c()
  short_severe = c()
  long_severe = c()
  n_sessions = c()

  for(i in seq_along(data[,1])){
    #print(i)
    pad = 0
    sessions = getSessions(i,"a",drinking_only = T,data = data)
    sessions$date = as_date(sessions$date)
    inten.sessions =  sessions[1:n_session,]$intake
    freq.sessions = as.Date(sessions[1:n_freq,]$date)

    if(all(is.na(freq.sessions))){
      freq.sessions = c()
    }else{
```

```
      freq.sessions = na.omit(freq.sessions)
    }
    intensity[i] = mean(inten.sessions,na.rm=T)
    n = length(freq.sessions)
    total = intensity[i] * n
    if(n > 0){
      duration = as.numeric(difftime(data$surveydate_a[i],min(freq.sessions), units = "days"))
      pad = (duration / n) * .5
      duration = duration + pad
    } else{
      duration = NA
    }
    n_sessions[i] = n
    average[i] = total/duration
    max[i] = max(inten.sessions,na.rm=T)
    frequency[i] = n/duration
  }
  max[is.infinite(max)] = NA
  average[is.infinite(average)] = NA
  frequency[is.infinite(frequency)] = NA
  max[data$drinker_a == 0] = 0
  average[data$drinker_a == 0] = 0

  #Class Risks
  for(i in seq_along(max)){
    risk = classRisk(average[i],max[i])
    short[i]<- risk$short
      long[i] <-risk$long
      short_severe[i] <- risk$short_severe
      long_severe[i] <- risk$long_severe
  }


  return(data.frame(list(n_sessions = n_sessions,max = max, intensity = intensity,
              average = average, frequency = frequency,
              short = short , long = long , short_severe = short_severe , long_severe = long_severe)))
}

#
```

Table 4. The total variance explained by shorter versions of the Finnish method in comparison to the full Finnish method

```
table4 = data.frame(matrix(nrow = 4, ncol = 4))
rownames(table4) = paste0("intensity_", 1:4)
names(table4) = paste0("freq_", 1:4)
for (c in seq_along(names(table4))) {
  for (r in seq_along(rownames(table4))) {
    #print(r)
    table4[r, c] = cor.test(f$averageintake_a,
                            shorten(r, c, data = f)$average,
                            method = "pearson")$estimate
```

```
  }
}
table4 = round(table4 ^ 2, 3) * 100
table4 %>%
  knitr::kable(booktabs = T,
               longtable = F,
               format = "latex")
```

	freq_1	freq_2	freq_3	freq_4
intensity_1	95.9	95.9	96.1	96.6
intensity_2	98.6	98.6	98.8	99.7
intensity_3	98.6	98.6	98.8	99.6
intensity_4	98.8	98.8	99.0	100.0

Table 4b. the total variance explained by shorter versions of the Finnish method in comparison to the full Finnish method - excluding cases with no variance between drinking occasions

In order to excluded cases of lazy reporting we repeated the analysis with cases of no variance excluded.

```
table4b = data.frame(matrix(nrow = 4, ncol = 4))
rownames(table4b) = paste0("intensity_", 1:4)
names(table4b) = paste0("freq_", 1:4)
for (c in seq_along(names(table4b))) {
  for (r in seq_along(rownames(table4b))) {
    #print(r)
    table4b[r, c] = cor.test(tr$averageintake_a,
                             shorten(r, c, data = tr)$average,
                             method = "pearson")$estimate
  }
}
table4b = round(table4b ^ 2, 3) * 100
table4b %>%
  knitr::kable(booktabs = T,
               longtable = F,
               format = "latex")
```

	freq_1	freq_2	freq_3	freq_4
intensity_1	97.9	97.9	98.3	100
intensity_2	97.9	97.9	98.3	100
intensity_3	97.9	97.9	98.3	100
intensity_4	97.9	97.9	98.3	100
